# Exposure to the Great Famine in Early Life and the Risk of Obesity in Adulthood: A Report Based on the China Health and Nutrition Survey

**DOI:** 10.3390/nu13041285

**Published:** 2021-04-14

**Authors:** Huiru Jiang, Yongfu Yu, Leah Li, Wanghong Xu

**Affiliations:** 1 Department of Epidemiology, School of Public Health, Fudan University, Key Laboratory of Public Health Safety, Ministry of Education Fudan University, 138 Yi Xue Yuan Road, Shanghai 200032, China; 18111020007@fudan.edu.cn; 2 Department of Biostatistics, School of Public Health, Fudan University, 138 Yi Xue Yuan Road, Shanghai 200032, China; yu@fudan.edu.cn; 3 Population, Policy and Practice Research and Teaching Department, University College London Great Ormond Street Institute of Child Health, London WC1N 1EH, UK; leah.li@ucl.ac.uk

**Keywords:** famine exposure, body mass index, waist circumference, overweight, central obesity, adulthood

## Abstract

Intrauterine malnutrition has a long-term effect on human health. This study aimed to evaluate the associations between exposure to famine in early life and obesity in adulthood in Chinese adults. A total of 5033 participants (22,132 observations) of the China Health and Nutrition Survey (CHNS) in 1991–2015 were classified into three famine exposure groups according to their birth year: unexposed (1963–1966), fetal-exposed (1959–1962) and childhood-exposed (1955–1958). Compared with the unexposed group, the fetal-exposed group had higher levels of body-mass-index (BMI) and waist-circumference (WC), and higher prevalence of overweight and central obesity, whereas the childhood-exposed group had lower levels of the measurements. However, the positive associations of fetal exposure with BMI, WC and prevalence of overweight and central obesity were attenuated by additionally adjusting for age at survey. Stratified analysis showed that the adverse effect of fetal exposure to famine was only observed in subjects at several specific age-groups, and in men living in rural areas and in women living in more severe famine exposed areas (*p* for interaction < 0.05). Our results provide evidence for the weak effect of fetal exposure to famine on body measurements in adulthood, and suggest the importance of severity of famine exposure and timing of exposure.

## 1. Introduction

The increase in overweight/obesity has attracted major attention around the world [1]. The global epidemic of the abnormal has been largely explained by increasing exposure to lifestyle risk factors such as physical inactivity and high-energy intake [2]. It is also well-established that exposure to adverse environments in early life, especially during the critical period of fetal growth, has a long-term effect on development of chronic diseases later in life, including overweight/obesity [3]. Intrauterine malnutrition has been associated with elevated susceptibility to obesity in adulthood [4,5,6].

Studies of the Dutch and Ukrainian famines have shown that prenatal exposure to famine, an extreme form of environmental stress, was associated with an increased risk of subsequent obesity [7,8]. Several other studies observed a positive famine exposure-–adulthood obesity association only in women but not in men [9,10,11]. A null [9,12] and even an inverse association [12] were also reported between famine exposure at certain stages of early life (i.e., early childhood, the last trimester of pregnancy and the first months of life) and subsequent obesity. Evidently, the results were far from consistent.

China experienced the Great Leap Forward Famine between 1959 and 1961 [13], which served as a “nature experiment” to investigate the potential effect of early-life exposure to famine on health in adulthood. However, the evidence on the potential influence of famine exposure on subsequent obesity is limited and conflicting [14,15,16,17,18,19,20]. While an increased risk of subsequent obesity was observed for subjects exposed to famine during fetal and childhood periods relevant to those born after the famine [16,17,18,19,20], no significant association was observed in other studies [14,15]. In studies based on the China Health and Nutrition Survey (CHNS) data, exposure to famine was consistently associated with increased risk of overall obesity [21,22] and central obesity [23]. However, these studies, either based on cross-sectional or longitudinal data, used birth date to define famine exposed and unexposed groups, namely, using the subjects born after the famine as the unexposed group. Due to the older age of the exposed groups than the unexposed group, the strong positive confounding effect of age may have biased the associations between fetal or childhood exposure to famine and obesity in adulthood away from the null. In a meta-analysis of observational studies, adjustments for age and other factors have been observed to nullify some associations of famine exposure with the risks of cardiometabolic conditions in adulthood [24].

In this study, we used the large-scale longitudinal data of the CHNS to examine the associations of famine exposure in early life with body measurements in adulthood, by adjusting for age at survey and conducting stratified analysis by age groups, which may minimize the potential confounding effect of age. We also evaluated the potential modifying effect of the severity of famine exposure in early life and socioeconomic status (SES) at survey on the associations.

## 2. Materials and Methods

### 2.1. Study Design and Subjects

The CHNS is an ongoing multipurpose longitudinal household survey, initiated in 1989 in nine provinces and two autonomous regions. A multistage random cluster sampling design was adopted in the survey, as described in previous studies [25]. Questionnaire and anthropometric data were collected in 1989, 1991, 1993, 1997, 2000, 2004, 2006, 2009, 2011, and most recently, in 2015. Considering that the 1989 survey followed a different standardized procedure, used different stadiometers or scales from those in subsequent surveys, and did not collect pregnant status for women [26], we included data collected between 1991 and 2015 and downloaded the data from the website of the program (https://www.cpc.unc.edu/projects/china) (accessed on 1 September 2020).

Ineligible observations, including those from Beijing, Shanghai and Chongqing, three municipalities added to the sampling framework of the survey since 2011, were excluded from this analysis (Figure S1). As a result, we included 22,132 observations from a total of 5033 subjects born in 1955 to 1966. As presented in Table S1, age at survey, BMI, WC, prevalence of overweight and central obesity were observed to increase along with calendar year of survey in both men and women.

Due to the nature of the longitudinal design of the survey, repeated measurements were performed for some subjects. In this study, the number of repeated measurements for participants ranged from 1 to 9 with a mean of 4. A total of 770 individuals had only 1 measurement, 843 had 2, 503 had 3, 521 had 4, 537 had 5, 557 had 6, 611 had 7, 440 had 8 and 251 had 9.

### 2.2. Measures

#### 2.2.1. Exposure to Famine

Similar to previous studies focusing on famine exposure, we used birth year to define exposure status to famine in early life. Since the Chinese Great Famine lasted for three years, from 1959 to 1961, most participants born in 1962 might have experienced the famine during fetal development. Therefore, we defined famine exposure subgroups as: (1) unexposed group (born in 1963 to 1966); (2) fetal-exposed group (born in 1959 to 1962); (3) childhood-exposed group (born in 1955 to 1958).

#### 2.2.2. Covariates

Residence of participants was classified into rural and urban areas according to 3-digit codes released by the National Bureau of Statistics of China, and were grouped by severity of famine exposure, which was determined according to total fertility loss during from 1958 to 1965 [27]. All provinces or autonomous regions having total fertility loss of 100% or over were defined more severe famine areas, while those with total fertility loss less than 100% were defined as less severe famine areas. In China, very few people moved from rural to urban areas or vice versa, or from one province or autonomous region to another, due to the policy of Hukou. Therefore, the residence of participants, either classified into rural or urban areas or grouped into more or less severe famine areas, may represent the famine exposure level of the subjects in early-life.

Socioeconomic index score (SEI) was used to measure SES of participants at the interview. SEI was calculated based on educational level, occupation and annual income per capita according to a scale for Chinese urban residents (version 2010) [28], adapted from the scale proposed by Duncan [29]. It was updated every year because income per capita changes with Consumer Price Index Numbers for Industrial Workers (CPI-IW) [30]. Educational level, occupation and annual income per capita were classified, scored and summarized as SEI score (Table S2). The SEI score was further classified into lower class (≤6) and upper class (>6) groups of SES.

#### 2.2.3. Outcomes

Body measurements were conducted following the standard protocol and techniques [31]. All participants were measured for standing height using a calibrated beam scale to the nearest 0.1 cm and body weight using a portable stadiometer to the nearest 0.1 kg with lightweight clothes and no shoes on. Waist circumference (to the nearest 0.1 cm, WC) was taken at the midpoint between the bottom of the rib cage and the top of the iliac crest at the end of exhalation.

Body mass index (BMI, kg/m^2^) was derived as measured body weight in kilograms divided by measured standing height in meters squared. Overweight was defined as BMI ≥ 25 kg/m^2^, and central obesity was defined as WC > 90 cm in men and >80 cm in women [32,33].

### 2.3. Statistical Analysis

Data were presented as mean ± standard deviation (SD) for continuous variables and frequency (percentage) for categorical variables. Comparisons of baseline characteristics across groups were conducted using Chi-squared tests or Analysis of Variance (ANOVA). The Cochran–Armitage test, Spearman’s rank-order correlation analysis and Kendall’s tau-b correlation analysis were used to test the trends. Taking repeated measurements for some participants into account, generalized linear mixed models were applied to estimate age-adjusted regression coefficients (β) and 95% confidence interval (CI) for famine exposure with BMI and WC, and age-adjusted odds ratios (OR) and 95%CIs for famine exposure with prevalent overweight and central obesity, to investigate the association of famine exposure with obesity risk. Stratified analyses were further conducted to estimate age-specific β (95%CI) and OR (95%CI) and evaluate the potential modifying effect of severity of famine exposure in early-life and SES measured at survey on the associations. 

Since the Chinese Great Famine happened during the period of 1959 to 1961, we conducted sensitivity analysis by redefining subjects born in 1959 to 1961 as the fetal-exposed group, and those born in 1955 to 1958 as the childhood-exposed group. The participants born in 1962 to 1966 were used as the unexposed group to make a comparison.

All analyses were performed using SAS version 9.4 (SAS Campus Drive, Cary, NC, USA) and R Studio version 4.0 (RStudio, Boston, MA, USA). *p* < 0.05 was considered as statistical significance.

## 3. Results

### 3.1. Characteristics of the Study Population

Table 1 shows the characteristics of participants by exposure to famine. The mean age at survey was 38.2 (SD: 7.8) years in the unexposed group, significantly younger than 41.4 (SD: 7.3) years in the fetal-exposed group and 44.0 (SD: 6.6) years in the childhood-exposed group. A significant difference was also observed for SES at survey and famine severity of residence across the three groups in both men and women. With regard to the body measurements, the fetal-exposed group had a higher average level of BMI and WC than the other two groups (*p* < 0.01). The prevalence of overweight and central obesity were also higher in the fetal-exposed group (*p* < 0.01).

### 3.2. Age-Specific Body Measurements by Exposure to Famine

**Figure 1:**
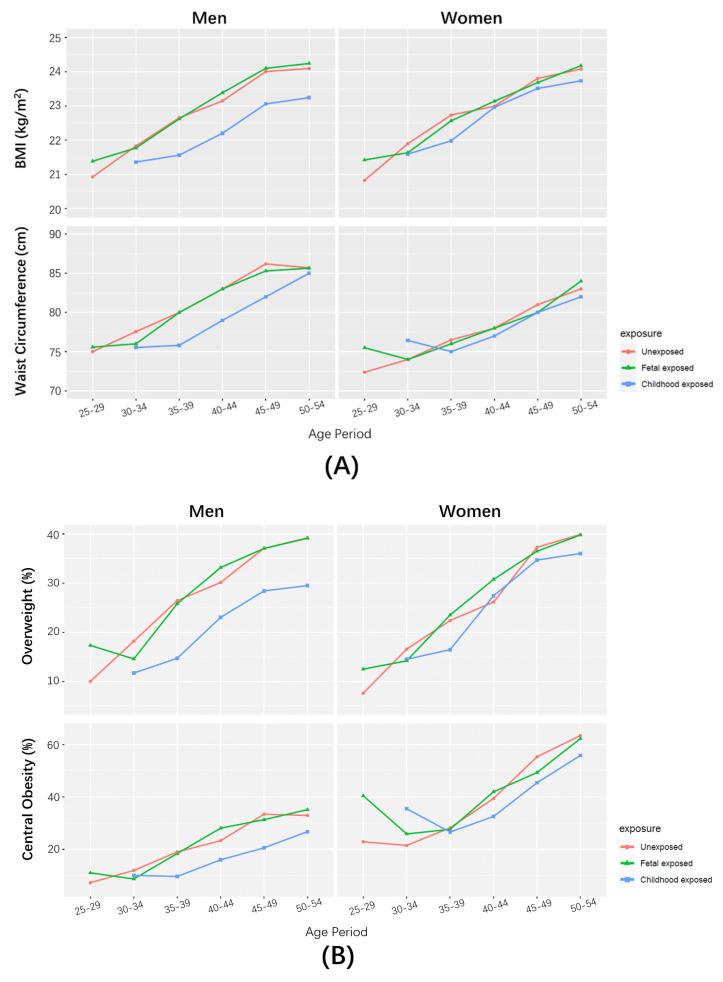
Body measurements at respective age periods by famine exposure. (**A**) Medians of BMI and WC. (**B**) Prevalence of overweight and central obesity.

Figure 1A shows the increasing body measurements with age in each subgroup of famine exposure. Generally, the average levels of BMI and WC in the fetal-exposed group were very close to those in the unexposed groups at each age period, but higher than those in the childhood-exposed group. The discrepancy with the childhood-exposed group appeared more evident in men than in women.

Similarly, the prevalence of overweight and central obesity also increased with age in each subgroup, and were higher in the fetal-exposed group and unexposed group than in the childhood-exposed group (Figure 1B).

### 3.3. Associations of Famine Exposure with Body Measurements in Adulthood

As shown in Table 2, BMI was 0.4 (95%CI: 0.1, 0.7) kg/m^2^ higher in the fetal-exposed men than in the unexposed men, but was 0.2 (−0.4, 0.1) lower in the childhood-exposed men. In women, a positive association was observed for both fetal- and childhood-exposed groups, with β (95%CI) being 0.3 (0.0, 0.6) and 0.3 (−0.0, 0.6), respectively. The positive associations in men and women, however, were reversed to be negative by additionally adjusting for age at survey. Similar association patterns before and after adjusting for age at survey were observed for famine exposure with WC. 

The significant positive associations between fetal exposure to famine with prevalent overweight and central obesity were also nullified by adjusting for age at survey, with age-adjusted OR (95%CIs) being 1.0 (0.8, 1.2) in men and 1.0 (0.8, 1.3) in women for prevalent overweight, and 1.0 (0.9, 1.2) in men and 1.0 (0.8, 1.1) in women for prevalent central obesity. Childhood exposure to famine was associated with reduced risks of overweight and central obesity, particularly after adjusting for age.

### 3.4. Associations of Famine Exposure with Body Measurements at Specific Age Periods

**Figure 2:**
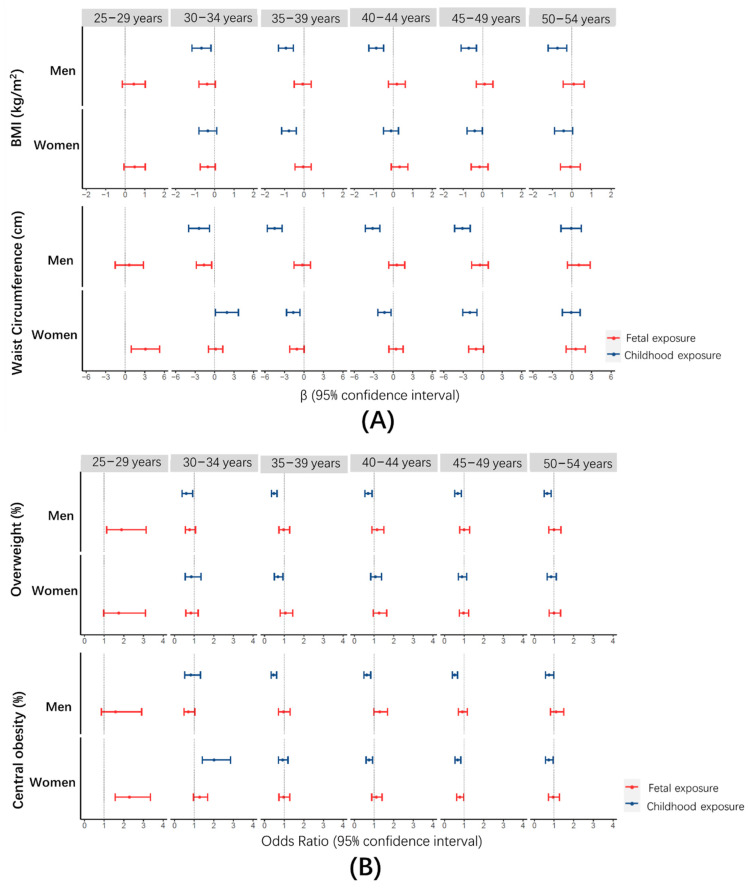
Associations of famine exposure with body measurements at respective age periods. (**A**) β and 95%CI for famine exposure with BMI and WC; (**B**) OR and 95%CI for famine exposure with prevalence of overweight and central obesity.

To exclude the confounding effect of age, we estimated the associations of famine exposure with body measurements at respective age periods. As shown in Figure 2A, a significant increase was only observed for WC at the age of 25–29 years in the fetal-exposed group (β: 3.1, 95%CI: 0.9, 5.3) relative to the unexposed group in women. Correspondingly, a significantly higher prevalence of central obesity was also found among these specific individuals (OR: 2.3, 95%CI: 1.5, 3.4) (Figure 2B). Compared with the unexposed groups, the childhood-exposed group was inversely associated with BMI, WC and prevalence of overweight and central obesity during most age periods.

### 3.5. Stratified Analysis of Associations between Famine Exposure and Body Measurements

We further evaluated the associations of famine exposure with body measurements by SES at survey and residences of subjects in early life. No significant interaction was found for SES with famine exposure on body measurements in adulthood (all *p* for interaction > 0.05). However, we observed a significant modifying effect of residences, either classified by areas or by severity of famine, on the associations. As shown in Table 3, the positive associations of fetal exposure to famine with BMI or prevalence of overweight were only observed in men living in rural areas (β: 0.2; 95%CI: −0.2, 0.5; OR: 1.1, 95%CI: 0.9, 1.4), and in women living in more severe famine areas (β: 0.3; 95%CI: −0.1, 0.7; OR: 1.3, 95%CI 0.9, 1.6), but not for those living in urban (β: −0.5, 95%CI: −1.0, 0.0; OR: 0.8, 95%CI: 0.6, 1.1) or less severe areas (β:−0.7, 95%CI: −1.2, −0.2; OR: 0.7, 95%CI: 0.5, 0.9) (*p* for interaction < 0.01). For the childhood exposure to famine, an inverse association was observed with body measurements regardless of urban or rural areas, or more or less severe famine areas.

### 3.6. Sensitivity Analysis

Sensitivity analysis observed similar results. As presented in Table S3, the fetal-exposed group had higher levels of BMI and WC, and higher prevalence of overweight and central obesity in both sexes, compared with the unexposed group. Additional adjusting for age at survey attenuated or even reversed the associations. 

## 4. Discussion

In this longitudinal study with a large sample size of Chinese adults, we observed higher levels of BMI and WC and higher prevalence of overweight and central obesity in the fetal-exposed group than in the unexposed group. However, the positive associations of fetal exposure to famine with body measurements were attenuated and even reversed by adjusting for age, and in stratified analysis by age groups. As a result, we only observed significant positive associations in specific age groups and among subjects exposed to more severe famine in early-life.

The “weak” effect of fetal exposure to famine observed in this study was somewhat inconsistent with previous studies, including those conducted in the Chinese population [16,17,23]. Almost all previous studies conducted in China were based on cross-sectional design and defined the exposure groups using the birth years of the subjects. Due to that, famine-exposed groups were several years older than those unexposed, age at survey became the most important confounder and might have led to overestimated positive associations. In this study, we adjusted age at body measurements to minimalize its confounding effect and revealed the nullified effect of famine exposure in early life.

In this study, we did not find significant a sex difference in the association pattern of famine exposure with body measurements. The results were somewhat inconsistent with many previous studies, in which women were observed more likely to be affected by famine [17,21,23]. Roseboom et al. [34] attributed the sex difference to the lower percentage of boys born alive due to fetal exposure to famine. Tobi et al. [35] explained the sex difference using the sex-specific altered DNA methylation and various phenotypes caused by famine exposure in utero. The “strong” adverse effect of the more severe and much longer famine in China may have overshadowed the existing “weak” sex discrepancy, and may help to explain the inconstancy of our results with those from other populations.

Our findings on the modifying effect of residence, an index for severity of famine exposure in early life, are consistent with previous studies, in which a higher risk of overweight/obesity was observed in subjects living in provinces experiencing more severe famine [36] and in rural areas [15,21]. Our results indicate the importance of severity of famine exposure and the timing of exposure, and provide further evidence on the limited effect of famine exposure.

In this study, we did not find that the associations between fetal exposure to famine and body measurements differed by SES at survey. The results were not consistent with previous studies [16,23] and the “thrifty phenotype” hypothesis [5]. The adverse effects of early exposure to nutritional deprivation have been suggested to be most exaggerated when faced with over-nutrition in later life [37]. The significant negative associations of famine exposure in childhood with body measurements were also inconsistent with previous studies [16,20]. A longer-term follow-up survey for our subjects is warranted, to confirm our results and to understand the mechanisms underlying the inconsistencies.

Our study has several strengths. First, our subjects came from a larger-scale nation-wide survey, which covered the majority of regions throughout China and provided a representative sample at national level. Second, the large sample size of the study enables us to run stratified analysis to control for the confounding effect of age and evaluate potential interactions. Finally, we classified study participants according to areas or famine severity of their residences, and adopted SEI to assess their SES at survey, which makes it possible to evaluate the potential joint effect of adverse exposures in early life and in adulthood.

Several limitations should be mentioned. First, similar to some previous studies [16,38,39], we used the birth year of our subjects to define subgroups of famine exposure. Considering that the Chinese Great Famine lasted for three years but lacks exact starting and ending dates, our definition may have introduced misclassification bias. Therefore, we ran a sensitivity analysis by adjusting the definition and did not observe substantially changed results, which partly mitigated the concern. Second, the average age of the fetal-exposed group was around 41 years at survey. Given that the prevalence of overweight began to rise among Chinese adults from 50 years of age [40], the sample size for the elders may not be big enough to evaluate the associations of fetal exposure to famine with body measurements at later adulthood. Long-term observation is warranted to evaluate the effect. Finally, we did not adjust for physical activity, diet and other lifestyle factors in this analysis, which may have biased our results. However, these lifestyle behaviors may not be strongly associated with famine exposure in early life, and thus might have no confounding effect in the analysis.

In conclusion, our analysis demonstrates a weak adverse effect of fetal exposure to famine on body measurements in adulthood and a protective effect of childhood exposure on obesity. The more pronounced effect of fetal exposure in subjects living in rural areas or more severe famine exposed areas indicates the importance of severity of famine exposure and the timing of exposure.

## Figures and Tables

**Table 1:** Characteristics of observations by exposure to the Great famine.

	Unexposed (*n* = 8859)	Fetal-Exposed (*n* = 6203)	Childhood-Exposed (*n* = 7070)	*p* Values
**Men**				
No. of observations	4503	3024	3602	
Age (years)	38.2 ± 7.8	41.4 ± 7.3	44.0 ± 6.6	<0.01
Areas of residence				0.28
Urban area	1445 (32.1)	1011 (33.4)	1211 (33.6)	
Rural area	3058 (67.9)	2013 (66.6)	2391 (66.4)	
Famine severity of residence				<0.01
Less severe	1525 (33.9)	1166 (38.6)	1189 (33.0)	
More severe	2978 (66.1)	1858 (61.4)	2413 (67.0)	
SES at survey				<0.01
Lower class	2029 (45.1)	1154 (38.2)	1777 (49.3)	
Upper class	2474 (54.9)	1870 (61.8)	1825 (50.7)	
BMI (kg/m^2^)	22.9 ± 3.6	23.3 ± 3.7	22.7 ± 3.4	<0.01
WC (cm)	81.2 ± 11.5	82.3 ± 11.2	80.8 ± 10.9	<0.01
Overweight	1154 (25.6)	891 (29.5)	834 (23.2)	<0.01
Central obesity	912 (20.3)	719 (23.8)	645 (17.9)	<0.01
**Women**				
No. of observations	4356	3179	3468	
Age (years)	39.0 ± 7.6	41.7 ± 7.4	44.6 ± 6.5	<0.01
Residence				0.94
Urban area	1420 (32.6)	1025 (32.2)	1120 (32.3)	
Rural area	2936 (67.4)	2154 (67.8)	2348 (67.7)	
Famine severity of residence				<0.01
Less severe	1468 (33.7)	1119 (35.2)	1068 (30.8)	
More severe	2888 (66.3)	2060 (64.8)	2400 (69.2)	
SES at survey				<0.01
Lower class	2552 (58.6)	1867 (58.7)	2402 (69.3)	
Upper class	1804 (41.4)	1312 (41.3)	1066 (30.7)	
BMI (kg/m^2^)	23.0 ± 3.3	23.3 ± 3.5	23.3 ± 3.4	<0.01
WC (cm)	78.0 ± 10.0	78.9 ± 10.2	79.1 ± 10.1	<0.01
Overweight	1070 (24.6)	911 (28.7)	990 (28.6)	<0.01
Central obesity	1641 (37.7)	1326 (41.7)	1426 (41.1)	<0.01

Data presented as number (%) for categorical variables and mean ± SD for continuous variables. Overweight defined as BMI ≥ 25 kg/m^2^, and central obesity defined as WC >90 cm in men and >80 cm in women. BMI: body mass index; WC: waist circumference.

**Table 2:** Associations of exposure to famine with body measurements in Chinese men and women.

	BMI (kg/m^2^)		WC (cm)		Overweight		Central Obesity	
	Unadjusted β (95%CI)	Age-Adjusted β (95%CI)	Unadjusted β (95%CI)	Age-Adjusted β (95%CI)	Unadjusted OR (95%CI)	Age-Adjusted OR (95%CI)	Unadjusted OR (95%CI)	Age-Adjusted OR (95%CI)
Men								
Fetal-exposed	0.4 (0.1, 0.7)	−0.0 (−0.3, 0.3)	1.2 (0.3, 2.0)	−0.5 (−1.3, 0.3)	1.2 (1.1, 1.4)	1.0 (0.8, 1.2)	1.2 (1.1, 1.5)	1.0 (0.9, 1.2)
Childhood-exposed	−0.2 (−0.4, 0.1)	−0.9 (−1.1, −0.6)	−0.3 (−1.1, 0.4)	−3.2 (−4.0, −2.5)	0.9 (0.7, 1.1)	0.6 (0.5, 0.7)	0.9 (0.7, 1.1)	0.6 (0.5, 0.7)
Women								
Fetal-exposed	0.3 (0.0, 0.6)	−0.0 (−0.3, 0.3)	0.9 (0.1, 1.6)	−0.3 (−1.1, 0.5)	1.2 (1.1, 1.5)	1.0 (0.8, 1.3)	1.2 (1.1, 1.4)	1.0 (0.8, 1.1)
Childhood-exposed	0.3 (−0.0, 0.6)	−0.4 (−0.7, −0.1)	1.1 (0.3, 1.8)	−1.3 (−2.0, −0.5)	1.2 (1.1, 1.5)	0.8 (0.7, 1.1)	1.2 (1.1, 1.3)	0.8 (0.7, 0.9)

Unexposed men and women used as reference groups. BMI: body mass index; WC: waist circumference; OR: odds ratio; 95%CI: 95% confidence interval.

**Table 3:** Associations of exposure to famine with body measurements in Chinese men and women by socioeconomic status (SES), residential area and severity of famine.

	Men				Women			
	BMI (kg/m^2^)	WC (cm)	OverWeight	Central Obesity	BMI (kg/m^2^)	WC (cm)	OverWeight	Central Obesity
**By SES**								
**Lower Class**								
Fetal-exposed	0.0 (−0.4, 0.4)	−0.7 (−1.8, 0.4)	1.0 (0.7, 1.3)	1.0 (0.7, 1.3)	−0.1 (−0.5, 0.3)	−0.4 (−1.4, 0.6)	0.9 (0.7, 1.2)	1.0 (0.8, 1.2)
Childhood-exposed	−0.7 (−1.0, −0.4)	−3.0 (−4.0, −2.1)	0.6 (0.4, 0.8)	0.6 (0.4, 0.7)	−0.6 (−0.9, −0.2)	−1.4 (−2.4, −0.5)	0.8 (0.6, 1.0)	0.8 (0.7, 1.0)
**Upper Class**								
Fetal-exposed	−0.1 (−0.4, 0.3)	−0.5 (−1.4, 0.5)	1.0 (0.8, 1.2)	1.0 (0.8, 1.2)	0.1 (−0.3, 0.4)	−0.2 (−1.2, 0.8)	1.2 (0.9, 1.5)	1.0 (0.8, 1.2)
Childhood-exposed	−0.8 (−1.1, −0.4)	−2.7 (−3.7, −1.8)	0.7 (0.6, 0.9)	0.6 (0.5, 0.8)	−0.4 (−0.8, 0.0)	−1.5 (−2.5, −0.5)	0.9 (0.7, 1.1)	0.7 (0.5, 0.8)
P for interaction	0.93	0.86	0.77	0.89	0.76	0.51	0.12	0.10
**By areas of residence**								
**Urban areas**								
Fetal-exposed	−0.5 (−1.0, 0.0)	−1.6 (−3.0, −0.2)	0.8 (0.6, 1.1)	0.7 (0.5, 0.9)	−0.1 (−0.5, 0.4)	−0.6 (−1.8, 0.7)	1.2 (0.8, 1.6)	1.0 (0.8, 1.3)
Childhood-exposed	−1.0 (−1.5, −0.5)	−3.3 (−4.7, −1.8)	0.5 (0.4, 0.8)	0.5 (0.3, 0.6)	−0.4 (−0.8, 0.1)	−0.6 (−1.8, 0.6)	0.9 (0.7, 1.3)	0.9 (0.7, 1.2)
**Rural areas**								
Fetal-exposed	0.2 (−0.2, 0.5)	0.1 (−0.9, 1.1)	1.1 (0.9, 1.4)	1.2 (0.9, 1.5)	−0.0 (−0.4, 0.4)	−0.2 (−1.1, 0.8)	1.0 (0.8, 1.3)	1.0 (0.8, 1.2)
Childhood-exposed	−0.8 (−1.1, −0.5)	−3.3 (−4.2, −2.4)	0.6 (0.5, 0.8)	0.6 (0.5, 0.8)	−0.5 (−0.8, −0.1)	−1.6 (−2.6, −0.6)	0.8 (0.6, 1.0)	0.7 (0.6, 0.9)
P for interaction	<0.01	<0.01	<0.01	<0.01	0.78	0.07	0.70	0.36
**By famine severity of residence**								
**Less severe**								
Fetal-exposed	−0.1 (−0.5, 0.4)	−0.8 (−2.1, 0.5)	1.0 (0.7, 1.4)	1.0 (0.7, 1.4)	−0.7 (−1.2, −0.2)	−2.1 (−3.3, −0.8)	0.7 (0.5, 0.9)	0.7 (0.5, 0.9)
Childhood-exposed	−0.8 (−1.3, −0.3)	−2.9 (−4.2, −1.5)	0.7 (0.5, 0.9)	0.6 (0.4, 0.8)	−0.4 (−0.9, 0.2)	−1.2 (−2.6, 0.2)	0.9 (0.7, 1.3)	0.7 (0.6, 1.0)
**More severe**								
Fetal-exposed	0.1 (−0.3, 0.4)	−0.1 (−1.2, 0.9)	1.0 (0.8, 1.3)	1.0 (0.8, 1.2)	0.3 (−0.1, 0.7)	0.7 (−0.3, 1.6)	1.3 (0.9, 1.6)	1.2 (0.9, 1.4)
Childhood-exposed	−0.9 (−1.2, −0.6)	−3.5 (−4.4, −2.5)	0.6 (0.5, 0.7)	0.5 (0.4, 0.7)	−0.5 (−0.9, −0.2)	−1.4 (−2.3, −0.4)	0.8 (0.6, 0.9)	0.8 (0.7, 0.9)
P for interaction	0.36	0.03	0.69	0.98	<0.01	<0.01	<0.01	<0.01

Data presented as β (95%CI) for increases in BMI and WC, and as OR (95%CI) for the risk of overweight and central obesity. All β and OR adjusted for age. BMI: body mass index; WC: waist circumference; OR: odds ratio; 95%CI: 95% confidence interval.

## Data Availability

Data described in the manuscript can be accessed at https://www.cpc.unc.edu/projects/china.

## Supplementary Materials

The following are available online at https://www.mdpi.com/article/10.3390/nu13041285/s1, Figure S1: Flow chart for selection of study participants, Table S1: Characteristics of observations by waves of survey, Table S2: Scoring of components for socioeconomic index, Table S3: Sensitivity analysis for associations between famine exposure and body measurements in Chinese men and women.
