# Supplementary material for: Exposure to the Great Famine in Early Life and the Risk of Obesity in Adulthood: A Report Based on the China Health and Nutrition Survey
Source: Nutrients. 2021 Apr 14;13(4):1285. doi: 10.3390/nu13041285 (PMC8070734; doi:10.3390/nu13041285)
Supplement: Supplementary file 1 [file nutrients-13-01285-s001.pdf]

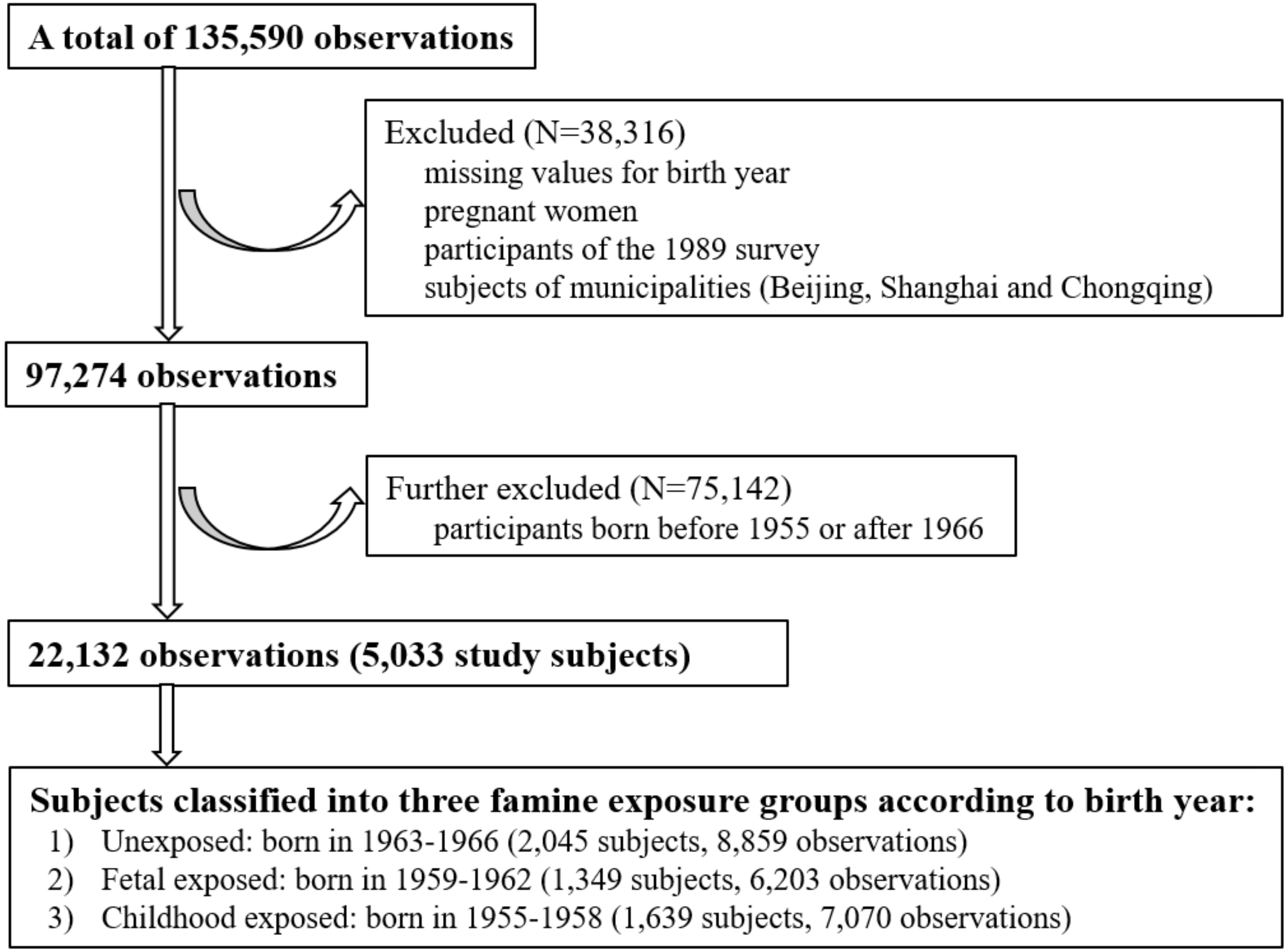

Figure S1: Flow chart for selection of study participants.

Table S1 Characteristics of observations by waves of survey

	Survey									*p for trend*
	1991 (n=2,656)	1993 (n=2,469)	1997 (n=2,567)	2000 (n=2,916)	2004 (n=2,637)	2006 (n=2,685)	2009 (n=2,747)	2011 (n=2,172)	2015 (n=1,283)	
**Men (n)**	**1424**	**1317**	**1314**	**1526**	**1277**	**1292**	**1334**	**1035**	**610**	
Age, years, mean ± sd	30.5±3.6	32.5±3.6	36.5±3.5	39.5±3.6	43.5±3.6	45.5±3.6	48.5±3.6	49.3±2.9	51.4±1.6	*<0.01*
Exposure to famine (n, %)										*<0.01*
Unexposed	545 (38.3)	497 (37.7)	499 (38.0)	578 (37.9)	477 (37.3)	481 (37.2)	503 (37.7)	478 (46.2)	445 (72.9)	
Fetal-exposed	358 (25.1)	332 (25.2)	353 (26.9)	420 (27.5)	348 (27.3)	349 (27.0)	355 (26.6)	344 (33.2)	165 (27.1)	
Childhood-exposed	521 (36.6)	488 (37.1)	462 (35.1)	528 (34.6)	452 (35.4)	462 (35.8)	476 (35.7)	213 (20.6)	0	
Areas of residence (n, %)										*0.76*
Urban	476 (33.4)	405 (30.8)	442 (33.6)	500 (32.8)	431 (33.8)	431 (33.4)	445 (33.4)	353 (34.1)	184 (30.2)	
Rural	948 (66.6)	912 (69.3)	872 (66.4)	1026 (67.2)	846 (66.2)	861 (66.6)	889 (66.6)	682 (65.9)	426 (69.8)	
Famine severity of residence										*<0.01*
Less severe	397 (27.9)	372 (28.3)	546 (41.5)	545 (35.7)	469 (36.7)	453 (35.1)	485 (36.4)	388 (37.5)	225 (36.9)	
More severe	1027 (72.1)	945 (71.7)	768 (58.5)	981 (64.3)	808 (63.3)	839 (64.9)	849 (63.6)	647 (62.5)	385 (63.1)	
Socioeconomic status (n, %)										*<0.01*
Lower class	809 (56.8)	750 (57.0)	627 (47.7)	704 (46.1)	621 (48.6)	530 (41.0)	451 (33.8)	308 (29.8)	160 (26.2)	
Upper class	615 (43.2)	567 (43.0)	687 (52.3)	822 (53.9)	656 (51.4)	762 (59.0)	883 (66.2)	727 (70.2)	450 (73.8)	
BMI, kg/m^2, mean ± sd	21.5±3.3	21.7±3.3	22.3±3.4	23.0±3.6	23.3±3.4	23.6±3.4	23.7±3.5	24.1±3.3	24.3±3.9	*<0.01*
WC, cm, mean ± sd	75.8±11.3	76.1±9.6	78.9±10.1	81.1±11.1	82.9±10.2	83.8±10.1	85.1±10.4	86.8±10.6	85.9±12.8	*<0.01*
Overweight (n, %)	171 (12.0)	175 (13.3)	262 (19.9)	415 (27.2)	367 (28.7)	399 (30.1)	449 (33.7)	388 (37.5)	253 (41.5)	*<0.01*
Central obesity (n, %)	140 (9.8)	94 (7.1)	172 (13.1)	299 (19.6)	288 (22.6)	322 (24.9)	380 (28.5)	365 (35.3)	216 (35.4)	*<0.01*
**Women (n)**	**1232**	**1152**	**1253**	**1390**	**1350**	**1393**	**1413**	**1137**	**673**	
Age (years, mean ± sd)	30.6±3.5	32.5±3.5	36.4±3.6	39.6±3.6	43.5±3.5	45.3±3.6	48.4±3.5	49.2±2.9	51.4±1.6	*<0.01*
Exposure to famine (n, %)										*<0.01*
Unexposed	433 (35.1)	416 (36.1)	463 (37.0)	482 (34.7)	492 (36.2)	537 (38.6)	527 (37.3)	534 (47.0)	472 (70.1)	
Fetal-exposed	365 (29.6)	335 (29.1)	357 (28.5)	393 (28.3)	378 (27.8)	378 (27.1)	399 (28.2)	373 (32.8)	201 (29.9)	
Childhood-exposed	434 (35.3)	401 (34.8)	433 (34.5)	515 (37.0)	490 (36.0)	478 (34.3)	487 (34.5)	230 (20.2)	0	

Areas of residence (n, %)										*0.42*
Urban	407 (33.0)	364 (31.6)	432 (34.5)	447 (32.2)	438 (32.2)	438 (31.4)	460 (32.5)	374 (32.9)	205 (30.5)	
Rural	825 (67.0)	788 (68.4)	821 (65.5)	943 (67.8)	922 (67.8)	955 (68.6)	953 (67.5)	763 (67.1)	468 (69.5)	
Famine severity of residence										*<0.01*
Less severe	307 (24.9)	295 (25.6)	504 (40.2)	468 (33.7)	468 (34.4)	474 (34.0)	490 (34.7)	409 (36.0)	240 (35.7)	
More severe	925 (75.1)	857 (74.4)	749 (59.8)	922 (66.3)	892 (65.6)	919 (66.0)	923 (65.3)	728 (64.0)	433 (64.3)	
Socioeconomic status (n, %)										*<0.01*
Lower class	861 (69.9)	819 (71.1)	826 (65.9)	893 (64.2)	928 (68.2)	880 (63.2)	774 (54.8)	567 (49.9)	273 (40.6)	
Upper class	371 (30.1)	333 (28.9)	427 (34.1)	497 (35.8)	432 (31.8)	513 (36.8)	639 (45.2)	570 (50.1)	400 (59.4)	
BMI, kg/m², mean ± sd	21.6±2.7	21.8±2.7	22.7±3.4	23.2±3.3	23.5±3.4	23.6±3.4	24.0±3.5	24.3±3.7	24.5±3.5	*<0.01*
WC, cm, mean ± sd	75.7±11.1	73.4±7.8	75.7±9.2	77.4±9.0	79.2±9.1	80.0±9.3	81.8±9.6	82.9±10.5	83.2±11.1	*<0.01*
Overweight (n, %)	143 (11.6)	147 (12.8)	264 (21.1)	362 (26.0)	413 (30.4)	437 (31.4)	493 (34.9)	439 (34.9)	273 (40.6)	*<0.01*
Central obesity (n, %)	434 (35.2)	190 (16.5)	319 (25.5)	441 (31.7)	561 (41.3)	626 (44.9)	740 (52.4)	654 (57.5)	428 (63.6)	*<0.01*

Overweight defined as BMI≥25 kg/m², and central obesity defined as WC>90 cm in men and >80 cm in women.

Table S2 Scoring of components for **s**ocioeconomic index

Socioeconomic components	Classification	Score
**Educational level**		
	College or above	6 [a]
	High school	4
	Middle school	3
	Elementary school	2
	Illiterate or semiliterate	1
**Occupation**		
	Senior professional/manager/technical	7
	Junior professional/manager/technical	6
	Administrator	5
	Office staff	4
	Skilled worker	3
	Manual worker	2
	Unemployed	1
**Annual income *per capita* (USD)**		
In 1991 survey		
	≥ 3825	7
	2550 ~ 3824	6
	1913 ~ 2549	5
	1276 ~ 1912	4
	638 ~ 1275	3
	319 ~ 637	2
	≤ 318	1
In 1993 survey		
	≥ 4644	7
	3096 ~ 4643	6
	2323 ~ 3095	5
	1549 ~ 2322	4
	775 ~ 1548	3
	387 ~ 774	2
	≤ 386	1
In 1997 survey		
	≥ 7683	7
	5122 ~ 7682	6
	3842 ~ 5121	5
	2562 ~ 3841	4
	1281 ~ 2561	3
	640 ~ 1280	2
	≤ 639	1
In 2000 survey		
	≥ 7444	7
	4963 ~ 7443	6
	3722 ~ 4962	5
	2482 ~ 3721	4
	1241 ~ 2481	3
	620 ~ 1240	2
	≤ 619	1
In 2004 survey		
	≥ 7956	7

	5304 ~ 7955	6
	3978 ~ 5303	5
	2653 ~ 3977	4
	1327 ~ 2652	3
	663 ~ 1326	2
	≤ 662	1
In 2006 survey		
	≥ 8263	7
	5509 ~ 8262	6
	4132 ~ 5508	5
	2755 ~ 4131	4
	1378 ~ 2754	3
	688 ~ 1377	2
	≤ 687	1
In 2009 survey		
	≥ 9253	7
	6169 ~ 9252	6
	4627 ~ 6168	5
	3085 ~ 4626	4
	1543 ~ 3084	3
	771 ~ 1542	2
	≤ 770	1
In 2011 survey		
	≥ 10789	7
	7193 ~ 10788	6
	5395 ~ 7192	5
	3597 ~ 5394	4
	1799 ~ 3596	3
	898 ~ 1798	2
	≤ 897	1
In 2015 survey		
	≥ 11598	7
	7735 ~ 11597	6
	5801 ~ 7734	5
	3868 ~ 5800	4
	1934 ~ 3867	3
	966 ~ 1933	2
	≤965	1

[a] According to Li's criteria, educational attainment of college or above including junior college (score 5), senior college (score 6) and graduate school (score 7). Due to lack of the details, an average score of 6 used for the group.

Table S3 Sensitivity analysis for associations between exposure to famine and body measurements in Chinese men and women

	BMI (kg/m²)		WC (cm)		Overweight		Central Obesity	
	Unadjusted β (95%CI)	Age-adjusted β (95%CI)	Unadjusted β (95%CI)	Age-adjusted β (95%CI)	Unadjusted OR (95%CI)	Age-adjusted OR (95%CI)	Unadjusted OR (95%CI)	Age-adjusted OR (95%CI)
Men								
Fetal-exposed	0.1 (-0.2, 0.5)	-0.3 (-0.6, 0.1)	0.2 (-0.7, 1.1)	-1.4 (-2.3, -0.5)	1.1 (0.9, 1.3)	0.9 (0.7, 1.1)	1.1 (0.9, 1.3)	0.8 (0.7, 1.0)
Childhood-exposed	-0.3(-0.5, -0.0)	-0.9 (-1.2, -0.7)	-0.7 (-1.5, -0.0)	-3.4 (-4.2, -2.7)	0.8 (0.7, 0.9)	0.6 (0.5, 0.7)	0.8 (0.7, 0.9)	0.5 (0.5, 0.6)
Women								
Fetal-exposed	0.2 (-0.1, 0.5)	-0.2 (-0.5, 0.2)	0.5 (-0.4, 1.3)	-0.7 (-1.6, 0.1)	1.2 (1.0, 1.5)	1.0 (0.8, 1.3)	1.1 (1.0, 1.3)	0.9 (0.8, 1.1)
Childhood-exposed	0.2 (-0.1, 0.5)	-0.5 (-0.8, -0.2)	0.8 (0.1, 1.6)	-1.4 (-2.1, -0.6)	1.2 (1.0, 1.4)	0.8 (0.7, 1.1)	1.1 (1.0, 1.3)	0.8 (0.7, 0.9)

Unexposed men and women used as the reference groups
